# The Validation of a New Visual Anaemia Evaluation Tool HemoHue HH1 in Patients with End-Stage Renal Disease

**DOI:** 10.1155/2013/424076

**Published:** 2013-04-08

**Authors:** Robert M. Kalicki, Stefan Farese, Dominik E. Uehlinger

**Affiliations:** Department of Nephrology, Hypertension and Clinical Pharmacology, Inselspital Bern, University Hospital and University of Bern, Freiburgstrasse 15, 3010 Bern, Switzerland

## Abstract

In chronic haemodialysis patients, anaemia is a frequent finding associated with high therapeutic costs and further expenses resulting from serial laboratory measurements. HemoHue HH1, HemoHue Ltd, is a novel tool consisting of a visual scale for the noninvasive assessment of anaemia by matching the coloration of the conjunctiva with a calibrated hue scale. The aim of the study was to investigate the usefulness of HemoHue in estimating individual haemoglobin concentrations and binary treatment outcomes in haemodialysis patients. A prospective blinded study with 80 hemodialysis patients comparing the visual haemoglobin assessment with the standard laboratory measurement was performed. Each patient's haemoglobin concentration was estimated by seven different medical and nonmedical observers with variable degrees of clinical experience on two different occasions. The estimated population mean was close to the measured one (11.06 ± 1.67 versus 11.32 ± 1.23 g/dL, *P* < 0.0005). A learning effect could be detected. Relative errors in individual estimates reached, however, up to 50%. Insufficient performance in predicting binary outcomes (ROC AUC: 0.72 to 0.78) and poor interrater reliability (Kappa < 0.6) further characterised this method.

## 1. Introduction

Anaemia is a feature commonly encountered in daily medical practice especially in well-defined clinical subpopulations such as nephrologic, oncologic, or pediatric patients. Diagnosis and therapeutic monitoring of anaemia are based on blood sampling and laboratory measurements, both necessitating the presence of qualified personnel, logistic, and technical resources and generate high costs especially if repetitive measurements are required. This is particularly striking when considering end-stage renal disease patients treated with recombinant human erythropoietin (rHuEPO) [1]. The imperative to reach and stay within a narrow haemoglobin concentration target range [2, 3], the peculiar pharmacokinetic and pharmacodynamic properties of rHuEPO [4–8], making its use difficult even in hands of experienced nephrologists, has led to the general acceptance of systematic and frequent monitoring of the haemoglobin concentration levels during therapy with rHuEPO. 

Although in developed countries laboratory facilities are easily accessible, the availability of a simple, cheap, noninvasive, and reproducible bedside method to assess the degree of anaemia in patients necessitating serial measurements would be very suitable [9, 10].

Severe anaemia may be detected by the naked-eye in the presence of significant skin pallor, pale nail beds and palms, whereas the examination of the conjunctiva provides in general a more sensitive and accurate estimation independent of the skin pigmentation [9–13]. However, this method remains crude and largely observer-dependent since the intensity of the conjunctiva colour is not matched with a reference hue [10].

**Figure 1:**
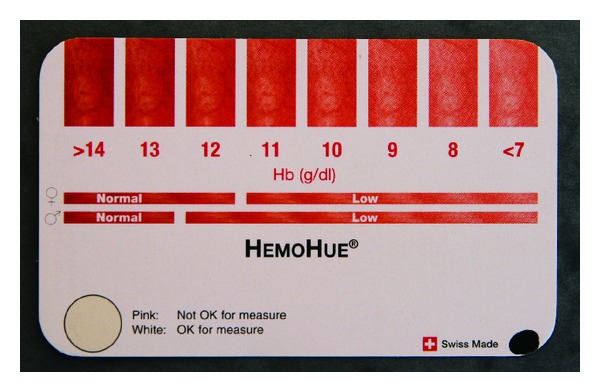
HemoHue HH1, HemoHue Ltd. Credit card sized device with an imprinted red hue, corresponding Hb levels, and a white luminescent control spot.

The HemoHue HH1 device (HemoHue Ltd) consists of a credit card-like small tool with an imprinted red hue consisting in seven red spots with increasing colour intensity matched with increasing haemoglobin concentrations (Figure 1).

The aim of the present prospective, comparative, and blinded study was to assess the validity of the HemoHue HH1 device in detecting and scaling the degree of anaemia in chronic haemodialysis patients. Eventual effects of an increased observer practice, formation, and general characteristics such as age and gender, on estimation of the haemoglobin concentration were evaluated. The ability of this new method in correctly detecting patients' haemoglobin concentrations inside and outside the therapeutic range was also assessed.

## 2. Subjects and Methods

### 2.1. Patients

Eighty chronic haemodialysis patients from our dialysis ward were enrolled in the study. Inclusion criteria were age over eighteen and capacity to understand the aim of the study and to give verbal consent. Any of the following excluded the patient from participating in study: acute or chronic affection of the anterior segment of the eye and the unavailability of a laboratory measurement of haemoglobin within two weeks of the visual assessment or the presence of a clinically relevant bleeding and/or transfusion requirements. The study was approved by the local ethics committee board, Kantonale Ethikkommission Bern, Universität Bern, Switzerland.

### 2.2. Study Design and Methods

Patients were assessed in decubitus or semidecubitus position on the dialysis chair within the first two hours of their usual treatment session. Localization of the patient in the dialysis room in relation to the natural light intensity (next to the window, intermediary, next to the door), the day time, and the ultrafiltration performed at the time of measurement was recorded. Two commercially available polychromatic neon tubes providing natural light, fixed perpendicularly on a rolling tripod, were used to ensure optimal lighting conditions as indicated by the whitening of the control luminescent spot on the HemoHue HH1 card.

The visual estimation of the haemoglobin concentration was performed as follows: the inferior lid was retracted, and the most intensely colored spot of the conjunctival sack was compared with the colored spots on the HemoHue HH1 card and matched. This procedure was repeated independently by all seven observers: one medical student (*med stud*), one dialysis nurse (*nurse*), three physicians with increasing age and degree of clinical experience (*phys A, phys B, and phys C*), and two administrative employees (*desk A and desk B*). The sequence in which the estimations were performed was randomized each time. Every observer was blinded for the estimates of all coobservers and the measured haemoglobin values during the whole study.

The procedure mentioned above was repeated on two nonconsecutive haemodialysis sessions at a two-week interval (*1st and 2nd session*). In the meantime, the laboratory measurement of haemoglobin was performed in all subjects at the beginning of a haemodialysis session, hence providing one individual reference value. Some observers participated to both sessions and were, therefore, considered to be *skilled* as opposed to those who assisted only punctually to the first or the second session (*novice*).

### 2.3. Statistics

The software package Systat 12 (SPSS Inc., Chicago, IL) and R.2.8.1 (R Development Core Team) were used for statistical analyses and graphical presentation. Values are given as mean ± standard deviation (SD) if not indicated otherwise. Intergroup analysis was performed by one-way ANOVA with Bonferroni post-hoc analysis. The package “irr” v. 0.7 (R Development Core Team) was used to compute the Cohen's and Fleiss' Kappa test [14] for the interobserver reliability. The overall agreement between the visual and the standard method was assessed with the Bland-Altman plot [15]. 

According to our preliminary statistical analysis, 68 patients were necessary to reject the null hypothesis in discriminating between both methods (measured haemoglobin-estimated haemoglobin concentration = ±5 g/L) to reach a power of 90% at a significance level of 5%.

## 3. Results

From the initially evaluated 80 patients during the first session, 75 were still available for visual estimations during the second session. Five dropouts were recorded (3 absences and 2 missing laboratory data). Patient characteristics are summarized in Table 1. 

### 3.1. Pooled Data Analysis

The estimated population mean was close to the measured one (11.06 ± 1.67 versus 11.32 ± 1.23 g/dL, *P* < 0.0005), and the slight underestimation could be improved during the second session as indicated by the reduced absolute residuals (estimated and measured Hb concentration, *1st session* versus *2nd session*:  −0.47 ± 0.07  versus  −0.06 ± 0.07 g/dL, *P* < 0.0005). 

**Figure 2:**
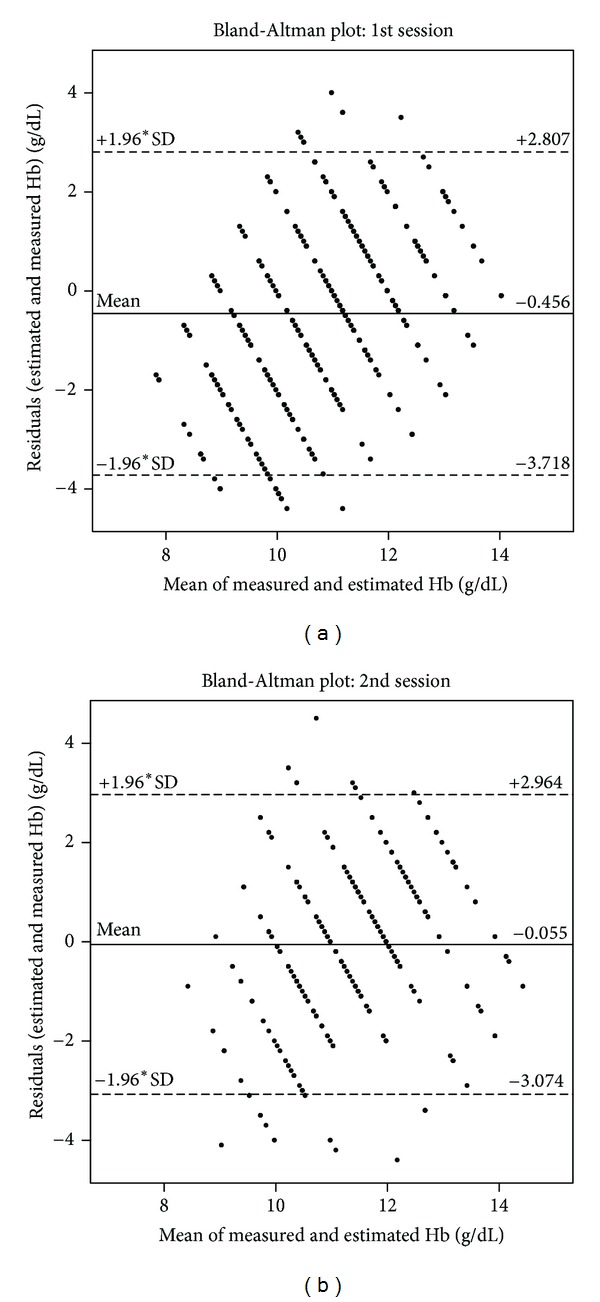
Bland-Altman plot highlighting the results of the first (a) and second (b) session. On the *X*-axis, is plotted the mean of the measured and estimated Hb values. The absolute residuals (estimated and measured) are plotted on the *Y*-axis. The plain line represents the mean residual, the mean ±1.96 ∗ SD is represented by the dotted lines, respectively.

The analysis of the Bland-Altman plots (mean of estimated and measured haemoglobin values versus absolute residuals consisting of estimated and measured values) provided further information concerning the performance of this method (Figure 2). As may be easily seen from these plots, a poor agreement between the estimated and the measured values was found, highlighting a clear systematic error (misspecification) with underestimation in the low concentration range and overestimation in the higher range. In accordance with the previous statement, the linear regression performed on pooled values yielded a rather flat line with a high intercept:  [Hb]_Measured_ = 0.302 · [Hb]_Estimated_ + 7.984  in [g/dL];  *R* = 0.1692,  *P* < 0.001. Further, for the same measured value, very high dispersion of the estimates could be noticed with relative error reaching nearly 50%. 

### 3.2. Interobserver Differences and Multivariate Analysis

Dichotomizing the observers into two groups: medical student and physicians (*physicians*) versus nurse and administrative employees (*nonphysicians*) permitted to show an improved accuracy of the estimate in the *physicians* versus the *nonphysicians* group (ΔResidual = 0.626 g/dL, *P* < 0.0005). *Skilled* observers gave a more accurate estimation of Hb concentrations as compared with *novices* (ΔResidual = 0.341 g/dL, *P* < 0.001). Crossing both categories provided further significant results for all subcategories with the exception of the “*physicians *× *skilled”* versus “*physicians *×* novice*” pair. 

Cofactors interfering with the lighting conditions such as the position of the patient in the dialysis room in relation to the windows or the day time did not influence significantly the accuracy of the estimates. The same was observed if the estimates were corrected by the actual amount of ultrafiltration at the time of measurement. A trend towards better estimates in younger patients with presumed less degenerative conjunctival affections (<*40 years*) compared to older ones (>*80 years*) (+0.032 versus −0.544 g/dL, *P* = 0.064) as well as the providing of higher estimates by the two last observers due to the hyperthermic effect of the manipulation on the conjunctiva (*observers* [1–5] *versus observers* [6, 7]) (−0.327 versus −0.127 g/dL, *P* = 0.066) were noted. The correction with the confounding factor *(observer)* failed, however, in achieving the significance level. The gender of the patients and the observers was not associated with any difference in the accuracy of the estimates.

### 3.3. Test Specificities and Interrater Agreement in Binary Outcomes

**Figure 3:**
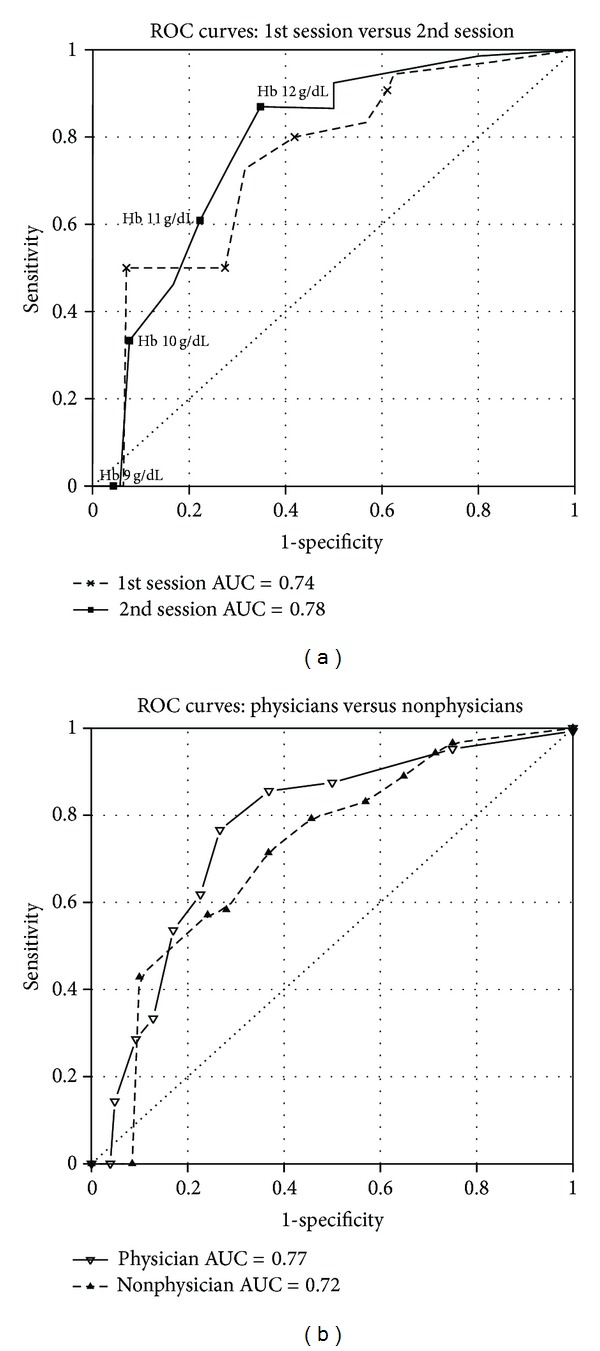
Receiver operating characteristic (ROC) curves for detecting anaemia at different cutoff values. First versus second session with corresponding haemoglobin concentration cutoff values and ROC AUC (a). Physicians versus nonphysicians and corresponding ROC AUC (b).

Besides the aptitude of the HemoHue HH1 tool to improve visual estimation of individual haemoglobin values, we, furthermore, tested its performance in providing estimation of binary treatment outcomes. According to KDOQI guidelines, the therapeutic haemoglobin target for rHuEPO treated patients should be in the range of 11.0 to 12.0 g/dL [2, 16]. Since the proposed range is very narrow and reaches the discrimination limit of the HemoHue HH1 device of ±1 g/dL, we arbitrarily defined the outcomes (*undertreatment*) as a haemoglobin concentration below 10 g/dL, (*within therapeutic range*) as a haemoglobin concentration between 10 and 12 g/dL, respectively, whereas (*overtreatment*) was set above 12 g/dL. In Table 2, the test specificities (sensitivity, specificity, positive and negative predictive values) and the interobserver agreement (reliability) in detecting the three scenarios described above are shown. As may be easily seen, the predictive performance of the method was slightly better at extreme haemoglobin values, especially in correctly rejecting *undertreatment*, without reaching, however, the standards to qualify as a good screening test. These findings are visually summarized with the receiver operating characteristic (ROC) curves in Figure 3. Furthermore, the reported values across subgroups (1st versus 2nd session, physician versus non-physician) were not substantially different. A slight to moderate interobserver agreement (reliability) for the outcome *undertreatment* and *overtreatment* could be noted (Kappa's ranging from 0.29 to 0.58), whereas a striking poor reliability could be observed for the *within therapeutic range* outcome. 

## 4. Discussion

To our knowledge, this is the first clinical trial which systematically assesses the validity of the novel visual bedside tool HemoHue HH1 for the estimation of haemoglobin concentration in chronic haemodialysis patients. 

Despite its simple use as a noninvasive and inexpensive test, convincing theoretical aspects and practical experience from previous clinical trials, this study clearly demonstrated the poor performance of the HemoHue HH1 tool in predicting visually the actual haemoglobin concentration in dialysis patients. This failure is evidenced by (1) the inability to estimate the actual haemoglobin level accurately based on one-observer guess, (2) no further consistent improvement of the accuracy when averaging the estimates of different observers, and finally (3) poor performance in estimating the binary treatment outcomes.

Although the estimated population mean was rather close to the measured population mean, individual estimates (one-observer estimation of one patient value) were crude with relative error ranging up to 50%. Further, as pointed out by the Bland-Altman plot, the presence of a systematic error with underestimation in lower Hb range and overestimation in the higher range was found. 

In the analysis of variance, a leaning effect with decreased residuals during the second session could be detected. The same feature was seen when comparing skilled with novice observers. It appears, however, improbable that this effect could be further substantially extended with practice. Assuming that the provided estimation is a sum of the true estimated value and an error which is largely due to interobserver discrimination aptitude, increasing the number of raters per item, should theoretically improve the precision of the estimates by lowering the noise component due to the error. However, this approach is hampered by the relative poor gain in accuracy over the individual approach and the difficulties in clinical implementation.

The estimation of the binary treatment outcomes was also characterized by an overall poor performance, especially by a very poor interobserver agreement in the therapeutic range. The test specificities for binary outcomes at the haemoglobin level of 11 g/dL were comparable with those described in previous works in nonnephrologic patients [10, 11].

There are different possible explanations of this failure. First, the achieved haemoglobin values in this particular population are within a narrow therapeutic range of 1 to 2 g/dL. When considering the density plot of the measured haemoglobin values, most of the patients were within the therapeutic range of 11 to 12 g/dL. The HemoHue HH1 device based on a visual estimation with a discrimination power of 1 g/dL could be not precise enough in this setting. 

Further, the inclusion of nephrologists aware of treatment goals and outcomes provides certainly a bias. The analysis of the density plots of the estimated values shows a narrow curve centred at known treatment targets in nephrologists. The curves in nonmedical staff were more dispersed about the theoretical mean and left skewed (tendency to provide low values). This may be underlined by the fact that there was no statistically significant difference in estimates between skilled and novice medical observers.

On the other side, our study population consisting principally of aged, chronic haemodialysis patients with some degree of degenerative ocular affection and vascular dysfunction could account in part for the discrepancy between the estimated and the measured haemoglobin level. So far, the visual anaemia evaluation was principally assessed and validated in paediatric and gynaecologic patients as a raw screening test. Although not significant, a trend toward decreased accuracy of the estimates in older patients was noted.

There are several limitations to this study including among others the lack of a real training component. Indeed, the observers in our study were neither aware of real results nor could they train themselves against reference values. However, the effect of training on the use of the HemoHue HH1 reference card for the visual estimation of hemoglobin values was not the aim of this study. As a matter of fact, the study was designed to prevent a carryover effect of education on the outcome by not providing the testers with a feedback on their performance. According to the manufacturer, the device can be used even by untrained people and still produce good results provided that adequate lighting conditions are fulfilled as indicated by the whitening of the control spot. In other words, appropriate estimation would have been ensured by the correct matching of the hue between the card and the conjunctiva, primary implicating skills in discriminating colors, rather than by conditioning the response of the observer with a reference value. 

Finally, training observers against reference values in the dialysis setting would have been impracticable. The time required to obtain laboratory results is much too long to permit the required simultaneous comparison since time delay may preclude correct estimation due to hemoconcentration induced by ultrafiltration during the dialysis procedure.

When we designed the study, available preliminary data clearly confirmed the superiority of the HemoHue HH1 reference card over the naked-eye estimation, and we assumed to achieve a comparable increase in accuracy in hemodialysis patients.

Despite undeniable further improvement in estimating visually the haemoglobin level as compared with the crude, naked-eye assessment, this method failed in estimating with acceptable accuracy the individual haemoglobin level as well as the individual binary treatment outcomes. The validity of these findings should be, however, limited to the studied particular haemodialysis population and not preclude the further deployment and validation of this method in other clinical groups. 

## 5. Conclusions

Despite undeniable additional improvement in estimating visually the haemoglobin levels as compared with the crude, naked-eye assessment, this method failed in predicting with acceptable accuracy the individual haemoglobin level as well as the individual binary treatment outcomes.

## Figures and Tables

**Table 1:** Patients characteristics (pooled sessions) (*N* = 80 on 1st session and *N* = 75 on 2nd session).

Age, years	66.0 ± 14.4	(70*)		
Race, white versus black	76 : 4			
Gender, male versus female	47 : 33			
Measured hemoglobin level, g/dL	11.32 ± 1.23	(11.3*)	*Q*_25–75_ = [10.6; 12.1]	
Estimated haemoglobin level, g/dL (pooled)	11.06 ± 1.67	(11.0*)	*Q*_25–75_ = [10.0; 12.0]	*Q*_25–75_ = [10.21; 12.00]^†^

Values are mean ± SD.

*Indicates the median.

^†^Quartiles based on the mean of all observers per patient.

**Table 2:** Test specificities in detecting binary outcomes.

Outcome	Sens.	Spec.	PPV	NPV	Kappa	Sens.	Spec.	PPV	NPV	Kappa
	1st session					2nd session				

Hb < 10 g/dL	50	73	29	89	0.34	33	92	38	91	0.29
10 g/dl ≤ Hb ≤ 12 g/dL	53	48	60	42	0.22	74	56	70	62	0.31
Hb > 12 g/dL	39	91	58	82	0.45	65	87	71	83	0.46

	Physicians					Non-physicians				

Hb < 10 g/dL	32	87	29	88	0.44	55	76	29	90	0.39
10 g/dL ≤ Hb ≤ 12 g/dL	64	51	64	51	−0.01	59	46	60	45	−0.04
Hb > 12 g/dL	63	86	63	86	0.58	35	89	54	79	0.41

Sensitivity, specificity, and positive and negative predictive values are given in percents.
